# Perspectives from Dentists, Dental Assistants, Students, and Patients on Dental Care Adapted to the COVID-19 Pandemic: A Cross-Sectional Survey

**DOI:** 10.3390/ijerph18083940

**Published:** 2021-04-09

**Authors:** Maximiliane Amelie Schlenz, Alexander Schmidt, Bernd Wöstmann, Andreas May, Hans-Peter Howaldt, Dennis Albert, Doreen Ziedorn, Norbert Krämer, Nelly Schulz-Weidner

**Affiliations:** 1 Dental Clinic—Department of Prosthodontics, Justus Liebig University, Schlangenzahl 14, 35392 Giessen, Germany; alexander.schmidt@dentist.med.uni-giessen.de (A.S.); bernd.woestmann@dentist.med.uni-giessen.de (B.W.); 2 Department of Cranio-Maxillofacial Surgery, Justus Liebig University, Klinikstrasse 33, 35392 Giessen, Germany; andreas.may@dentist.med.uni-giessen.de (A.M.); hp.howaldt@uniklinikum-giessen.de (H.-P.H.); 3 Institute of Hygiene and Environmental Medicine, Justus Liebig University, Friedrichstrasse 16, 35392 Giessen, Germany; dennis.albert@uk-gm.de (D.A.); doreen.ziedorn@uk-gm.de (D.Z.); 4 Dental Clinic—Department of Pediatric Dentistry, Justus Liebig University, Schlangenzahl 14, 35392 Giessen, Germany; norbert.kraemer@dentist.med.uni-giessen.de (N.K.); nelly.schulz-weidner@dentist.med.uni-giessen.de (N.S.-W.)

**Keywords:** dentistry, health care, questionnaires, COVID-19, coronavirus, pandemics, patient care, dental students, dental assistants, dentists

## Abstract

Dental care has been affected by SARS-CoV-2 (COVID-19) worldwide. In contrast to other dental clinics, the Justus-Liebig-University Giessen (Germany) decided not to limit dental treatment to emergencies alone, but to continue dental care for all patients, with increased safety measures. As such, health care professionals may be exposed to additional physical and mental stress. The aim of this study was to assess the perspectives of all persons involved in dental care (dentists, dental assistants, students, and patients) regarding the aspects of safety measures, anxiety about self-infection and infecting others, and other prospects in the period March to December 2020 using a questionnaire. Data collection was performed between 14 December 2020 and 23 January 2021. A total of 35 dentists (response rate of 79.5%), 23 dental assistants (65.7%), 84 students (80%), and 51 patients (21.8%) completed the survey. The patients did not notice any changes in the care received. Dentists and dental assistants reported a higher workload due to additional safety measures. The majority of dentists, students, and patients agreed that normal patient care was maintained. One-third of dental assistants would have preferred emergency treatment alone and expressed significantly higher anxiety about COVID-19 infection than all other groups (*p* < 0.05). In conclusion, all groups showed a predominantly positive perspective on dental care, and anxiety about self-infection and infecting others was especially low. However, additional measures are time-consuming and compound daily patient care. This concept, based on well-established infection control, might be a viable proposal for current and future pandemics.

## 1. Introduction

Coronavirus disease 2019 (COVID-19), caused by the severe acute respiratory syndrome coronavirus 2 (SARS-CoV-2), has affected health care systems worldwide including dentistry, which still faces challenges in daily patient care [1]. By the end of January 2020, the World Health Organization (WHO) and the Center for Disease Control and Prevention (CDC) published recommendations for the prevention and control of COVID-19 for health care professionals (HCP) [2,3]. Furthermore, institutions like the American Dental Association (ADA) issued its recommendation in March 2020 for dentists nationwide to postpone elective dental procedures and focus on emergency dental care alone, in response to the rapid spread of COVID-19 across the United States [4,5]. Additionally, Meng et al. advised dentists to take strict personal protection measures and avoid or minimize treatments that can produce droplets or aerosols to prevent cross infections in dental care between HCP and patients [6].

The need for dental care is unquestioned and it is generally accepted that infection control in dental clinics is very high. For example, the established dental care concept according to the hygiene guidelines of the German Study Group for Hygiene in Dentistry [7] has also been proven to be effective for several other infectious diseases (e.g., tuberculosis). Moreover, the current COVID-19 pandemic is not expected to end in the near future; therefore, elective treatments cannot be postponed indefinitely. Furthermore, the dental clinic of the Justus Liebig University Giessen (JLU), located in the State of Hessen in the middle of Germany, has an inborn training mission for students to treat dental patients even in infectious situations. Based on these facts, the dental clinic of the JLU decided, at the beginning of the pandemic, in March 2020, not to solely limit dental treatment to emergency care, but to continue normal patient care with additional hygiene and protective measures. At this time, no specific recommendations for dental care in Germany existed. Therefore, our established dental care concept in the dental clinic was adapted to the new COVID-19 pandemic based on international guidelines and recommendations [3,4,6].

It is described in the literature that the professional environment may moderate or worsen mental health in the new pandemic scenario [8]. Furthermore, the new daily work routine in this health emergency has been reported to cause substantial physical, personal, and emotional distress including an increased risk of mental health issues [9,10,11,12,13,14,15]. Additionally, an increased workload associated with inadequate personal protection equipment (PPE), causing concerns of nosocomial transmission that may result in physical exhaustion, anxiety, and even stress [16,17,18,19,20]. Similar studies that only focused on dental assistants or students confirmed these results [21,22]. However, all groups dealing with patients, dentists, dental assistants, students, and patients have to follow a pandemic concept focusing on the well-being of all persons involved [23].

To the best of our knowledge, there is no study described in the literature that investigated the perspective of all involved persons in dental care toward a dental care concept adapted to COVID-19 to continue normal patient care and not limit dental treatment to sole emergencies.

Therefore, the aim of this cross-sectional survey was to assess the dental care concept of the JLU adapted to COVID-19 from the perspective of dentists, dental assistants, students, and patients regarding the aspects of safety measures, anxiety about self-infection and infecting others, and other prospects in the period March to December 2020. It was hypothesized that there is no difference between the four groups regarding the perspective on dental care adapted to the COVID-19 pandemic.

## 2. Materials and Methods

### 2.1. Dental Care Concept Adapted to COVID-19 Pandemic

Since the beginning of the COVID-19 pandemic in Germany in March 2020, the council of the dental clinic of the JLU has decided to continue normal patient care and not limit dental treatment to sole emergencies.

Therefore, in cooperation with the Institute of Hygiene and Environmental Medicine of the JLU, the established hygiene concept for dental clinics, based on the Robert Koch Institute [24] and the German Study Group for Hygiene in Dentistry [7], was expanded with additional safety measures including new registration and patient management processes, reconstruction, and personal protective equipment.

All groups (dentists, dental assistants, students, and patients) were made aware using information signs and internal and external communication from the departments. Wearing face coverings was made obligatory in the entire building. A controlled entry and exit system with a one-way system, and a questionnaire on health status and history of travel or contact to confirmed COVID-19, as recommended by Meng et al. [6], were established. People with common COVID-19 symptoms (fever, dry cough, and loss of taste and smell) and stayed in a risk area defined by the RKI within the last 14 days without a negative COVID-19 test were not allowed to enter the dental clinic. The same regulation applied to persons who have had contact to a confirmed COVID-19 case within the past 14 days or who have been quarantined by the health department. Additional hand disinfection for people entering the clinic was ensured by means of disinfectant dispensers, which were available at the main entrances and exits and in each department. Only a limited number of persons were allowed to stay in the registration and waiting areas, depending on the size (in square meters) of each room. Regarding this aspect, attention was paid to keep a distance in between individual people of at least 1.5 m and good ventilation. This was later defined as cross-ventilation every 20 min or after every sneeze and cough, or 2 min shock ventilation with wide-open window and open door, or 10 min with wide open window, or 60 min with tilted window. After each patient, treatment fallow time was considered for ventilation of the room. In addition, the use of washrooms, elevators, and locker rooms was limited with regard to the maximum allowed number of people in the room. Therefore, the number of students treating patients at the same time was halved compared to the time before the COVID-19 pandemic. Patients were encouraged to arrive on time (not too early or late) for their appointment to avoid congestion at entrances and exits, and thus, contact with each other. Waiting times were reduced to a minimum, meaning that patients were seated as soon as possible. Moreover, all appointments were made only via telephone.

All treatment halls of the student-patient courses were structurally partitioned into individual treatment rooms, and only the usage of dentists’ chairs with access to fresh air (window boxes) was allowed for patient care.

All HCP (dentists, dental assistants, and students) received PPE including N95/FFP2 respirators, gloves, and full-face shields. For aerosol-generating treatments, the use of a rubber dam was recommended. Since the beginning of the pandemic, a polymerase chain reaction (PCR) COVID-19 test has been conducted before surgical intervention. All dental staff and students were only tested when suspected of 2019-nCoV or presenting symptoms. Since mid-December 2020, the corona antigen rapid test has been used comprehensively. HCP are tested twice a week and patients are tested before aerosol-generating treatments.

For 2019-nCoV confirmed or suspected patients, a separate treatment room was set up on the ground floor (polyclinic) to avoid unnecessary contact.

### 2.2. Online Survey

Four online questionnaires (one each for dentists, dental assistants, students, and patients) were designed in cooperation with the Teaching Evaluation Service Center of the JLU and provided using an online survey tool (LimeSurvey, Hamburg, Germany, Supplementary Materials). The survey contained questions or statements regarding the way participants dealt with the dental care concept adapted to COVID-19 including additional safety measures, anxiety about becoming infected or infecting others, and future prospects. In addition, the anxiety to become infected with COVID-19 at the dental clinic was retrospectively enquired for the period March to December 2020. For systematic evaluation, a five-point Likert scale [25] was used for all questions and statements, and abstention was allowed. Finally, demographic questions were asked. The survey was evaluated anonymously, and the study was conducted in accordance with the ethical standards of the Institutional Review Board and the local ethics committee of the JLU (ref. no. 235/20).

All clinical dental students (semesters seven to 10) participating in the fall term of 2020/21 (*n* = 105), dentists (*n* = 44), and dental assistants (*n* = 35) involved in patient care were enrolled. Patients being treated in the dental clinic between 14 December 2020 and 22 January 2021, and who were regularly treated for at least two years (*n* = 234) were also asked to participate in this survey. While HCPs received a personal link to the online survey via email and received a reminder after seven and 28 days, the patients were able to participate by scanning a QR code at the registration desk within one day.

All participants were informed that the data collection was completely anonymous and did not allow any traceability of the person answering. Data collection was performed between 14 December 2020 and 23 January 2021. Only completed questionnaires were included. 

Statistical analyses were performed using SPSS Statistics (version 26; IBM, Armonk, NY, USA). The distribution of responses is presented as the mean and standard deviation. Furthermore, data on anxiety were analyzed. In detail, one-way ANOVA with groups as a principal factor was performed for anxiety analysis of self-infection and infection of other persons. Logistic regression analysis was conducted to analyze the general anxiety of COVID-19 infection in different groups with the following control variables: age, gender, and affiliation to a risk group. As no significant differences were observed between the groups, only data on general anxiety were presented. All data were corrected with Bonferroni tests for multiple testing (*p* < 0.05).

## 3. Results

**Figure 1:**
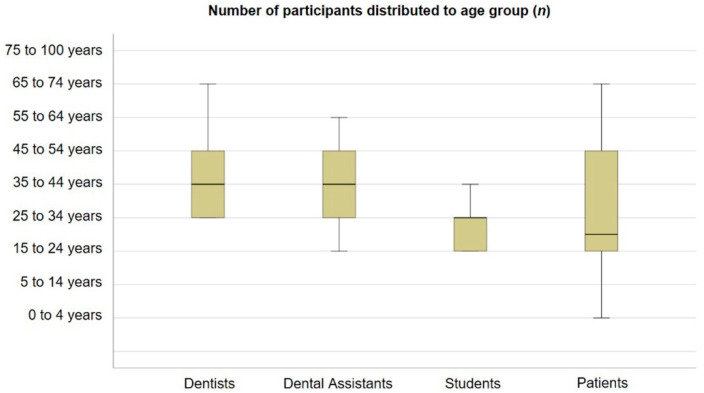
Number of participants distributed to age group (*n*).

A total of 35 (18 females, 11 males, six no answer) dentists completed the questionnaire, representing a response rate of 79.5%. The majority were aged 25–54 years (Figure 1). All dentists stated that they had direct contact with patients in their everyday work. Of the participants, 14.3% were at risk due to age (11.4%), chronic disease (5.7%), or obesity (2.9%). Only one dentist reported a confirmed COVID-19 infection while on vacation.

A total of 23 dental assistants (23 females, no males) responded to the survey, representing a response rate of 65.7%. The majority were aged 25–54 years (Figure 1). Fifteen dental assistants stated that they had direct patient contact, five reported indirect administrative patient contact, and three in other areas had no patient contact. Moreover, 56.5% counted themselves an at-risk group due to age (21.7%), chronic disease (21.7%), or obesity (4.3%). None of the patients had a history of COVID-19 infection. A total of 84 students (60 female, 20 male, one inter/trans, three no answer) completed the questionnaire, representing a response rate of 80%. The majority was distributed in the age group of 15 to 34 years (Figure 1). All had direct patient contact during their everyday work. Only two students counted themselves in the at-risk group due to immuno-suppression (1.2%), smoking (1.2%), obesity (1.2%), or blood disease (1.2%). Two students reported a COVID-19 infection in their medical history. A total of 51 patients (24 women, 20 men, one inter/trans, six no answer), consisting of 36 adults and 15 children, answered the questionnaire, representing a response rate of 21.8%. The majority of patients were aged between 15 and 54 years (Figure 1). Eleven patients (or their parents) were included in the at-risk group due to chronic disease (13.7%), age (11.8%), obesity (9.8%), smoking (3.9%), immunosuppression (2%), asthma (2%), tumors (2%), and epilepsy (2%). None of the patients had a history of COVID-19.

With respect to the dental care concept adapted to the COVID-19 pandemic, all groups stated that they felt sufficiently informed. Dentists and students were mainly informed internally by information from their department. Dental assistants felt mainly informed by external information, and patients, by information signs. While the majority of dentists, students, and patients agreed with the maintenance of normal patient care, one-third of dental assistants stated their preference to perform only emergency treatment (Appendix A, Table A1).

Regarding the reasonableness of the measures with regard to infection control (e.g., symptom questionnaires, controlled entry and one-way system, wearing face coverings, additional protective equipment (visors, N95/FFP2 masks, etc.), structural partitioning of the treatment boxes into individual ‘treatment rooms’, and additional hand hygiene) were considered reasonable by all groups (Likert scale ≥ 3.9). A COVID-19 test before each treatment was rated as less reasonable, especially by dentists (Likert scale 3.5) and dental assistants (Likert scale 3.7, Appendix A, Table A2).

In terms of time requirement, patients mentioned no change in waiting time for dental appointments, individual treatment sessions, or administrative processes. In contrast, dentists and dental assistants stated the need for more time for administration and organization of patient care, and time for individual treatment. Students noticed almost no change. In addition, the time for instrument processing (sterilization and disinfection) was considered the same by dental assistants (Appendix A, Table A3). Remarkably, patients perceived high compliance with hygiene rules in all areas, while dentists and dental assistants mentioned that hygiene compliance in the waiting rooms was sometimes missing (Appendix A, Table A3).

Considering possible impairments in additional infection control measures, the majority of dentists, dental assistants, and students agreed that they were affected by limited communication and lack of facial expressions due to additional protective equipment (e.g., visor, mask). The aspect of longer waiting time until the start of treatment by additional measures (e.g., filling out the questionnaire on health status and history of travel/contact) burdened the dental assistants. Most patients felt unaffected (Appendix A, Table A4).

On the question of whether participants in this survey were at least once afraid of being infected with COVID-19 during the stay in the dental clinic, six dentists, 15 dental assistants, 16 students, and four patients answered “yes”. The anxiety expressed by the dental assistants was significantly higher than that of students and patients (*p* < 0.05, Table 1).

In detail, dental assistants were significantly more afraid of infecting themselves than students and patients. Regarding the anxiety of infecting others, a significant difference could also be detected between dental assistants and patients (*p* < 0.05, Table 2). However, the majority in all groups stated that they did not feel uncomfortable while treating patients or receiving treatment within the dental care concept (Appendix A, Table A5).

**Figure 2:**
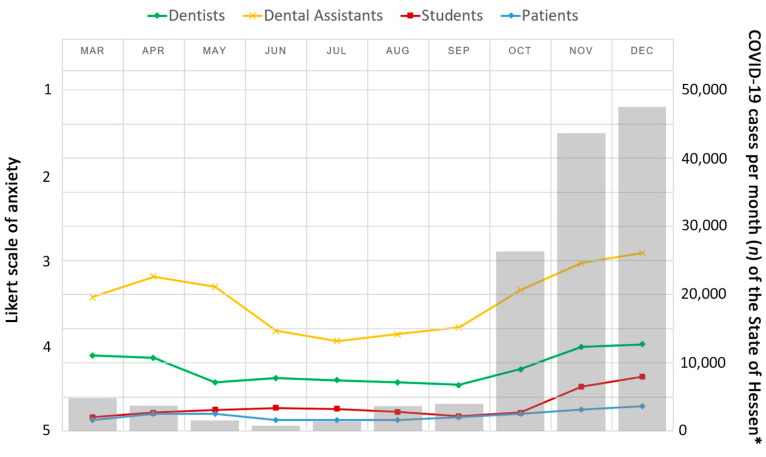
Schematic drawing of the COVID-19 cases per month in the state of Hessen from March to December 2020 (* according to the Robert Koch Institute [26]) (represented by bars) in relation to the reported anxiety by the four groups on the Likert scale (1 = great anxiety, 5 = no anxiety) to become infected with COVID-19 (represented by lines).

Figure 2 displays the anxiety of dentists, dental assistants, students, and patients to become infected with COVID-19 at the dental clinic in relation to the COVID-19 cases per month in the state of Hessen from March to December 2020. An impressive increase in anxiety was clearly demonstrated between October and December. Mainly dental assistants were affected, and the difference to students was significant (*p* < 0.05). A tendency of increased anxiety could be detected in the months of October, November, and December, always with the highest rate for dental assistants, and corresponding to the pandemic situation in the state of Hessen/Germany.

Different groups had different expectations about how long the COVID-19 pandemic would continue to hamper normal dental care, which were not obvious.

Regardless of this aspect, the majority of groups said that they would maintain additional protective measures after the COVID-19 pandemic with respect to other airborne infectious diseases (e.g., tuberculosis), depending on the situation (Table 3). Concerning the different protective measures, the most recommended items were special treatment rooms and the use of visors and N95/FFP2 masks from the practitioner’s end, followed by face covers throughout attendance, and hand hygiene (Table 3).

## 4. Discussion

The ongoing COVID-19 pandemic clearly emphasizes the necessity of reliable dental care concepts enabling normal dental care including dental check-ups, dental cleanings, and preventive care, because even these elective dental treatments cannot be postponed indefinitely. Aside from additional protective measures and equipment, health care professionals are urgently required, who feel safe and comfortable at their place of work.

To the best of our knowledge, the perspective of all involved persons in dental care (dentists, dental assistants, students, and patients) toward a dental care concept adapted to COVID-19 to continue normal patient care and not limit dental treatment to sole emergencies has not been described in the literature yet.

As all participants were informed that data collection was completely anonymous and did not allow any tracing, bias in answering was prevented. However, a clear limitation of this cross-sectional study is that the perspective of dentists, dental assistants, students, and patients from only one dental clinic at one time point were evaluated. It can only hypothesize, if different results would have found when conducting this survey earlier in the pandemic. For sure, there is more knowledge about COVID-19 today compared to the beginning of the pandemic, but there are still many uncertain surroundings. Therefore, it would be interesting to expand this survey to other countries and conduct the questionnaire study a second time at the end of this pandemic. Another limitation of this study is that the data collection was completed within one month. Given the possible rapid changes in regulations and infection rates, this short data collection period could affect the results. In particular, the retrospective question of the anxiety to become infected with COVID-19 at the dental clinic might not be so accurate. Therefore, the results should be interpreted with awareness of the retrospective character. The dental care concept adapted to COVID-19 was evaluated using a Likert scale, which is the standard procedure for surveys in the field of medicine [23,27,28,29,30].

Our approach was confirmed by the data of the presented survey, demonstrating high acceptance for dentists, students, and patients. Only one-third of the dental assistants diverged from this approach in their opinion to treat only emergencies. This may be due to the idea that all patients have to be considered as potentially infectious. These concerns are consistent with the idea of multiple dentists performing no dental procedures except emergencies [31]. Corresponding to the number of confirmed COVID-19 cases in Hessen, we found higher levels of anxiety regarding self-infection and infection of others in all groups, but the highest levels were found among dental assistants, even though all HCP worked at the same place and time during the pandemic.

There is no doubt that the concerns of the dental assistants may be due to their special situation: they have first-contact with potentially infected patients. Furthermore, they observe the non-compliance of patients with hygiene rules in the waiting rooms. A further factor may be insufficient manpower while performing daily work during the pandemic. This is important because even under normal conditions, an appropriate nurse–patient ratio is necessary [32].

Furthermore, gaps in protective hygiene measures, although recommended and established in our dental clinic, may be considered as a risk factor by our dental assistants [6].

HCP must be provided with sufficient PPEs including N95/FFP2 masks, gloves, isolation gowns, protective eye goggles, face shields, and head and shoes [33,34]. All the recommended PPEs have been implemented in our adapted concept. Moreover, these newly introduced measures are considered very useful by all groups investigated, despite restrictions in well-being and with regard to additional activities. Furthermore, there is wide consensus that these protective measures may continue after the abatement of the COVID-19 pandemic.

These aspects disprove our hypothesis, which expected no difference between the four groups regarding the perspective on dental care adapted to the COVID-19 pandemic.

In this context, it should be emphasized that the patients did not feel affected as a result of all the new procedures in the pandemic including having no concerns about the risk of a possible COVID-19 infection. However, a limitation of this study was the low response rate in the patient group. This may be due to the fact that patients were not invited by email with a personal address, but rather anonymously as part of the registration process, as this was the only option due to data protection reasons. In contrast to HCP groups, it was not possible to send the patient group a reminder. Therefore, patients had only one day to answer the questionnaire compared to HCPs. Since all participants could only fill out the questionnaire once, it can be assumed that no participant completed the questionnaire over several days, but at one point during the study period.

It can be discussed whether the participants had already adapted to the new measures because of the long investigation period between March to December 2020. The results of this survey indicate that HCP are particularly affected by the additional protective measures due to the pandemic and did not get used to the new procedures. 

Although the majority of public attention is focused on the probability of COVID-19 infection and its prevention in a dental setting, other health consequences resulting from people’s anxiety should be kept in mind [35]. As above-mentioned, dental assistants may be compromised in their work routine. However, 70% agreed with our newly adapted concept to not only treat emergencies, but also provide normal patient care. In our opinion, these results provide evidence of the high standards of the newly established dental care concept for COVID-19. It has proven to be successful and may be useful for future pandemics. We would like to also confirm these results at other dental clinics. 

## 5. Conclusions

This cross-sectional study provides evidence that the perspectives of dentists, dental assistants, students, and patients on dental care adapted to the COVID-19 pandemic were predominantly positive. Anxiety about self-infection or infecting others was low. However, additional protection following the dental care concept adapted to COVID-19 is time-consuming in daily patient care. This concept, based on a well-established infection control framework, might be a viable proposal for current and future pandemics.

## Figures and Tables

**Table 1:** Percentage of anxiety and results of odds ratios (OR) with 95% confidence interval (CI) for the pairwise comparisons (logistic regression analysis corrected by Bonferroni method).

	Anxiety (%)	OR (95% CI)/ *p*-Values			
		Dentists	Dental Assistants	Students	Patients
**Dentists**	27.3	-	6.31 (1.39–28.63)/ 0.102	0.62 (0.18–2.08)/ 1	0.24 (0.05–1.10)/ 0.396
**Dental Assistants**	78.9	-	-	0.10 (0.03–0.38)/ 0.006	0.04 (0.01–0.19)/ <0.001
**Students**	21.3	-	-	-	0.40 (0.10–1.58)/ 1
**Patients**	9.5	-	-	-	-
**Number of valid answers**		22	19	75	4

**Table 2:** Results of factorial variance analysis corrected by the Bonferroni method.

	*p*-Values							
	Afraid to Infect MYSELF.				Afraid to Infect OTHERS.			
	Dentists	Dental Assistants	Students	Patients	Dentists	Dental Assistants	Students	Patients
Dentists	-	0.445	0.721	>0.999	-	0.317	>0.999	>0.999
Dental Assistants	-	-	0.005	0.015	-	-	0.366	0.021
Students	-	-	-	>0.999	-	-	-	0.529

**Table 3:** Items regarding additional protective measures for future pandemics.

Item Description	Groups			
	Dentists (*n*)	Dental Assistants (*n*)	Students (*n*)	Patients (*n*)
Would you be in favor of maintaining the additional protective measures after the COVID-19 pandemic with regard to other airborne infectious diseases (e.g., tuberculosis)?				
Yes, always.	5	2	6	7
Yes, situation-dependent.	22	14	59	30
I cannot judge.	1	3	8	8
No.	7	3	10	6
What additional hygiene and protective measures would you recommend? (Multiple selection possible)				
Questionnaire on health status and history of travel/contact	15	10	39	21
One-way street system and entry system	10	7	18	13
Wearing face covering throughout the whole attendance	20	10	42	29
COVID-19 test before each treatment	3	4	21	6
Additional protective equipment (visors, N95/FFP2 masks etc.) on the practitioner’s side	22	13	50	28
Structural partitioning of the treatment boxes into individual “treatment rooms”	22	8	47	20
additional hand hygiene	20	12	46	29
Total number of participants	35	23	84	51

## Data Availability

The datasets of this article are available from the corresponding author on a reasonable request.

## Supplementary Materials

The following are available online at https://www.mdpi.com/article/10.3390/ijerph18083940/s1, Questionnaire 1: Questionnaire dental assistants; Questionnaire 2: Questionnaire dentists; Questionnaire 3: Questionnaire patients; Questionnaire 4: Questionnaire students.

## Appendix A

**Table A1:** Items and descriptive statistics about the information and acceptance of the dental care concept adapted to the COVID-19 pandemic.

Item Description	Groups							
	Dentists		Dental Assistants		Students		Patients	
	M (SD)	*N*	M (SD)	*N*	M (SD)	*N*	M (SD)	*N*
I Felt Sufficiently Informed about the Dental Care Concept Adapted to COVID-19 Pandemic, by…								
information signs. ^a^	3.6 (1.2)	34	3.7 (1.2)	22	4.1 (1.0)	80	4.4 (0.9)	51
internal information of the department (e.g., meetings, student council, online, personal information transfer). ^a^	4.1 (1.3)	34	3.6 (1.4)	22	4.2 (0.9)	80	4.0 (1.3)	46
external information of the department (e.g., university newsletter, webpage). ^a^	3.3 (1.2)	33	4.1 (0.9)	22	3.1 (1.3)	60	2.7 (1.6)	35
I Think It Is Good that Regular Patient Treatment is Maintained with Increased Hygiene and Protective Measures and not only Emergency Treatment is Provided. ^a^								
	4.2 (1.1)	34	3.4 (1.3)	23	4.7 (0.8)	82	4.9 (0.3)	51
Total number of participants		35		23		84		51

M, mean; SD, standard deviation; *N*, number of valid answers. ^a^ type of answer: 1 = strongly disagree, 5 = strongly agree.

**Table A2:** Items and descriptive statics about the reasonableness of additional infection control measures.

Item Description	Groups							
	Dentists		Dental Assistants		Students		Patients	
	M (SD)	*N*	M (SD)	*N*	M (SD)	*N*	M (SD)	*N*
How Reasonable do you Think the Following Measures are with Regard to Infection Control in the Dental Clinic?								
Questionnaire on health status and history of travel/contact ^b^	3.9 (1.3)	34	4.1 (1.1)	23	4.0 (1.2)	82	4.4 (1.1)	51
One-way system and entry system ^b^	4.3 (0.8)	35	4.0 (1.5)	23	3.9 (1.1)	82	4.4 (1.1)	50
Wearing face covering throughout the whole attendance ^b^	4.9 (0.2)	35	4.9 (0.4)	23	4.9 (0.3)	81	4.9 (0.7)	51
COVID-19 test before each treatment ^b^	3.5 (1.3)	35	3.7 (1.2)	22	3.9 (1.3)	82	3.8 (1.5)	49
Additional protective equipment (visors, N95/FFP2 masks etc.) on the treatment side ^b^	4.9 (0.2)	35	4.7 (0.7)	23	4.8 (0.6)	82	4.9 (0.5)	50
Structural partitioning of the treatment boxes into individual “treatment rooms ^b^	4.5 (0.8)	35	4.7 (0.9)	23	4.7 (0.7)	81	4.8 (0.8)	49
Additional hand hygiene ^b^	4.9 (0.4)	35	4.7 (0.8)	23	4.8 (0.6)	79	4.9 (0.4)	51
Total number of participants		35		23		84		51

M, mean; SD, standard deviation; *N*, number of valid answers. ^b^ type of answer: Likert scale: 1 = not reasonable; Likert scale: 5 = very reasonable.

**Table A3:** Items and descriptive statics about time requirement and compliance of hygiene rules.

Item Description	Groups							
	Dentists		Dental Assistants		Students		Patients	
	M (SD)	*N*	M (SD)	*N*	M (SD)	*N*	M (SD)	*N*
Please Assess whether and to What Extent the Time Requirement Has Changed since the COVID-19 Pandemic with Regard to the Following Aspects Compared to before the Pandemic.								
Waiting period for dental Appointment ^c^	-	-	-	-	-	-	1.9 (0.5)	41
Time for administration and organization of patient care ^c^	2.7 (0.4)	33	2.8 (0.5)	22	2.2 (0.7)	69	2.1 (0.6)	47
Duration in waiting room ^c^	-	-	-	-	-	-	1.7 (0.6)	48
Time for individual treatment Session ^c^	2.5 (0.5)	34	2.7 (0.5)	20	2.1 (0.6)	58	2.0 (0.4)	48
Time for instrument processing (sterilization and disinfection) ^c^	-	-	2.2 (0.4)	20	-	-	-	-
Do you Think, that All Hygiene Rules Related to Infection Control were Complied with in the Following Areas?								
Patient registration ^d^	3.9 (1.0)	32	4.0 (1.0)	22	4.4 (0.8)	66	4.7 (0.8)	51
Waiting Rooms ^d^	3.3 (1.1)	33	3.2 (1.2)	22	4.2 (0.9)	65	4.6 (0.7)	50
Treatment/dentist’s room ^d^	4.0 (1.0)	33	4.1 (1.0)	21	4.5 (0.8)	75	4.7 (0.6)	51
Total number of participants		35		23		84		51

M, mean; SD standard deviation; *N*, number of valid answers. ^c^ type of answer: 1 = less, 2 = unchanged, 3 = more; ^d^ type of answer: 1 = never, 5 = always.

**Table A4:** Items and descriptive statics about the impairments of additional infection control measures.

Item Description	Groups							
	Dentists		Dental Assistants		Students		Patients	
	M (SD)	*N*	M (SD)	*N*	M (SD)	*N*	M (SD)	*N*
The Following Additional Infection Control Measures Affected Me.								
limited communication due to additional protective equipment (e.g., visor, mask) ^a^	3.6 (1.3)	34	3.4 (1.3)	23	3.4 (1.3)	78	2.6 (1.5)	49
lack of facial expression due to additional protective equipment (e.g., visor, mask) ^a^	3.7 (1.3)	35	3.8 (1.3)	23	3.4 (1.3)	79	2.8 (1.6)	49
Longer waiting time until the start of treatment session due to additional measures (e.g., questionnaire on health status and history of travel/contact) ^a^	2.9 (1.3)	34	3.4 (1.0)	23	2.6 (1.2)	74	2.3 (1.3)	50
Total number of participants		35		23		84		51

M, mean; SD, standard deviation; *N*, number of valid answers. ^a^ type of answer: 1 = strongly disagree, 5 = strongly agree.

**Table A5:** Items and descriptive statics of anxiety of infection and feeling by dental treatment.

Item Description	Groups							
	Dentists		Dental Assistants		Students		Patients	
	M (SD)	*N*	M (SD)	*N*	M (SD)	*N*	M (SD)	*N*
To What Extent Do You Feel the Following Statements Apply to Current Patient Care during the COVID-19 Pandemic?								
I am afraid to infect MYSELF. **^a^**	2.7 (1.2)	34	3.3 (1.1)	23	2.3 (1.2)	82	2.3 (1.2)	51
I am afraid to infect OTHERS. ^a^	2.7 (1.4)	34	3.4 (1.0)	21	2.8 (1.2)	82	2.4 (1.3)	50
I feel uncomfortable treating patients/get treated (only for group patients). ^a^	2.1 (1.2)	33	2.5 (1.2)	22	1.8 (1.0)	78	1.5 (0.9)	50
In my opinion, the patients/dental staff (only for group patients) feel uncomfortable ^a^	2.1 (0.9)	33	2.4 (1.1)	23	2.3 (1.1)	77	1.7 (1.1)	49
Total number of participants		35		23		84		51

M, mean; SD, standard deviation; *N*, number of valid answers. ^a^ type of answer: 1 = strongly disagree, 5 = strongly agree.
