# Supplementary material for: Perspectives from Dentists, Dental Assistants, Students, and Patients on Dental Care Adapted to the COVID-19 Pandemic: A Cross-Sectional Survey
Source: Int J Environ Res Public Health. 2021 Apr 9;18(8):3940. doi: 10.3390/ijerph18083940 (PMC8069116; doi:10.3390/ijerph18083940)
Supplement: Supplementary file 1 [file ijerph-18-03940-s001.zip › Questionnaire dentists.pdf]

A1 I felt sufficiently informed about the dental care concept adapted to COVID-19 pandemic, by...
Please select the appropriate answer for each point:

| | strongly disagree | disagree | neutral | agree | strongly agree | no answer |
|---|---|---|---|---|---|---|
| information signs | ○ | ○ | ○ | ○ | ○ | ○ |
| internal department information (e.g. meetings, student council, online, personal information transfer meeting) | ○ | ○ | ○ | ○ | ○ | ○ |
| external department information (e.g. university newsletter, webpage) | ○ | ○ | ○ | ○ | ○ | ○ |

A2: Please assess whether and to what extent patient care in the dental clinic SINCE the COVID-19 pandemic with regard to the following aspects has changed compared to before the pandemic:

| | less | unchanged | more |
|---|---|---|---|
| time required for students organisation | ○ | ○ | ○ |
| time required for patient organisation in the departments | ○ | ○ | ○ |
| time required for treatments | ○ | ○ | ○ |
| time required for individual treatment sessions | ○ | ○ | ○ |

B1 To what extent do the following statements apply to you about current patient care during the COVID-19 pandemic?

Please select the appropriate answer for each point:

| | strongly disagree | disagree | neutral | agree | strongly agree | no answer |
|---|---|---|---|---|---|---|
| I think it is good that regular patient treatment is maintained with increased hygiene and protective measures and not only emergency treatment is provided | ○ | ○ | ○ | ○ | ○ | ○ |
| I am afraid to infect MYSELF. | ○ | ○ | ○ | ○ | ○ | ○ |
| I am afraid to infect OTHERS. | ○ | ○ | ○ | ○ | ○ | ○ |
| In my opinion, the patients feel uncomfortable. | ○ | ○ | ○ | ○ | ○ | ○ |
| I feel uncomfortable. | ○ | ○ | ○ | ○ | ○ | ○ |

B2. How useful do you think are the following measures with regard to protection against infection in the dental clinic/dental treatment?

| | strongly disagree | disagree | neutral | agree | strongly agree | no answer |
|---|---|---|---|---|---|---|
| questionnaire to query symptoms and stay in risk areas | ○ | ○ | ○ | ○ | ○ | ○ |
| one-way street and entry system | | | | | | |
| wearing mouth-to-nose coverings throughout the whole attendance | ○ | ○ | ○ | ○ | ○ | ○ |
| corona test before each treatment | ○ | ○ | ○ | ○ | ○ | ○ |
| additional protective equipment (visors, FFP2/N95 masks etc.) on the treatment side | ○ | ○ | ○ | ○ | ○ | ○ |
| additional hand hygiene | | | | | | |

B3. Which of the following aspects affect you?

| | strongly disagree | disagree | neutral | agree | strongly agree | no answer |
|---|---|---|---|---|---|---|
| limited communication due to additional protective equipment (e.g. visor, mask) | ○ | ○ | ○ | ○ | ○ | ○ |
| longer waiting time until the start of treatment session due to additional measures (e.g. COVID questionnaire) | ○ | ○ | ○ | ○ | ○ | ○ |
| lack of facial expression due to additional protective equipment (e.g. visor, mask) | ○ | ○ | ○ | ○ | ○ | ○ |

B4. Were you at least at one time afraid of being infected with COVID-19 at the dental clinic?

- Yes
- No

B5. Please indicate for each month how much fear you had of contracting COVID-19 at the dental clinic.

| | Large fear | rather fear | undecided | rather no fear | none fear |
|---|---|---|---|---|---|
| mar | ◯ | ◯ | ◯ | ◯ | ◯ |
| apr | ◯ | ◯ | ◯ | ◯ | ◯ |
| may | ◯ | ◯ | ◯ | ◯ | ◯ |
| jun | ◯ | ◯ | ◯ | ◯ | ◯ |
| jul | ◯ | ◯ | ◯ | ◯ | ◯ |
| aug | ◯ | ◯ | ◯ | ◯ | ◯ |
| sep | ◯ | ◯ | ◯ | ◯ | ◯ |
| oct | ◯ | ◯ | ◯ | ◯ | ◯ |
| nov | ◯ | ◯ | ◯ | ◯ | ◯ |
| dec | ◯ | ◯ | ◯ | ◯ | ◯ |

B6. Do you think, that all hygiene rules related to infection control were followed in the COVID-19 pandemic in the following areas?

| | never | sometimes | often | More often | always |
|---|---|---|---|---|---|
| patient registration | ◯ | ◯ | ◯ | ◯ | ◯ |
| waiting rooms | ◯ | ◯ | ◯ | ◯ | ◯ |
| treatment /Dentists´ room | ◯ | ◯ | ◯ | ◯ | ◯ |

B7. Do you perceive the increased hygiene rules and additional protective measures (e.g., wearing mouth/nose covering in the building, hand hygiene, keeping your distance, regular ventilation) as a burden during your stay at the dental clinic?

- Yes, the measures are a burden for me.
- No, the additional measures are not a problem for me.
- Others

C1. For how long (month/s) do you think the COVID-19 pandemic will continue to change our patient care in the dental clinic?

C2. Would you be in favor of maintaining the additional protective measures after the COVID-19 pandemic with regard to other airborne infectious diseases (e.g. tuberculosis)?

| | |
|---|---|
| Yes, always. | ○ |
| Yes, depending on the situation (e.g. in the winter months or when there is an increased risk). | ○ |
| I cannot judge | ○ |
| no | ○ |

C3. What additional hygiene and protective measures would you recommend? (Multiple selection possible)

| | |
|---|---|
| questionnaire to query symptoms and stay in risk areas. | ○ |
| one-way street system and entry system | ○ |
| wearing face coverings throughout the whole atttendance | ○ |

| | |
|---|---|
| Corona test before each treatment | ○ |
| additional protective equipment (visors, FFP2 masks, etc.) on the practioner´s side | ○ |
| structural partitioning of the treatment boxes into individual "treatment rooms" | ○ |
| additional hand hygiene | ○ |
| others | ○ |

D1. What is your gender?

Please select only one of the following answers:

- male
- female
- inter / trans
- 

D2. What age group do you belong to?

| | |
|---|---|
| 0- to 4-year-olds | ○ |
| 5- to 14-year-olds | ○ |
| 15- to 24-year-olds | ○ |
| 25- to 34-year-olds | ○ |
| 35- to 44-year-olds | ○ |
| 45 to 54-year-olds | ○ |
| 55- to 64-year-olds | ○ |
| 65- to 74-year-olds | ○ |
| 75 to 100-year-olds | ○ |

D3. In which area(s) are you predominantly active?

| | |
|---|---|
| organization (administrative tasks) | ○ |
| patient admission (e.g., outpatient clinic/poli) | ○ |
| patient treatment in the room/op | ○ |
| teaching with patient treatment (e.g. course supervision) | ○ |
| teaching without patient treatment (e.g. lecture, phantom classes) | ○ |
| science with patient contact | ○ |
| science without patient contact | ○ |
| others | ○ |

D4. Do you belong to the risk group with regard to a possible COVID-19 infection?

- Yes
- No

D5. Why do you belong to the risk group? (Multiple selection possible)

| | |
|---|---|
| Age +50 years | ○ |
| chronic disease (e.g. diabetes, high blood pressure, heart, lung, liver or kidney disease) | ○ |
| smoker | ○ |
| immunosuppression | ○ |
| overweight | ○ |
| pregnancy | ○ |

| | |
|---|---|
| others | ○ |

D6. Have you ever been diagnosed with COVID-19?

- Yes
- No

D7: Where do you think you became infected with COVID-19?

| | |
|---|---|
| in private environment (at home) | ○ |
| in private environment (vacation) | ○ |
| in professional environment (dental clinic) | ○ |
| in professional environment (dental praxis) | ○ |
| in professional environment (medical appointment) | ○ |
| in professional environment (stay in hospital ) | ○ |
| others | ○ |
