# Supplementary material for: Perspectives from Dentists, Dental Assistants, Students, and Patients on Dental Care Adapted to the COVID-19 Pandemic: A Cross-Sectional Survey
Source: Int J Environ Res Public Health. 2021 Apr 9;18(8):3940. doi: 10.3390/ijerph18083940 (PMC8069116; doi:10.3390/ijerph18083940)
Supplement: Supplementary file 1 [file ijerph-18-03940-s001.zip › Questionnaire patients.pdf]

A1 Please select, if you are a patient yourself or if your child is being treated by us.

- o I am a patient myself.
- o My child is a patient.

B1 I felt sufficiently informed about the dental care concept adapted to COVID-19 pandemic, by...

Please select the appropriate answer for each point:

	strongly disagree	disagree	neutral	agree	strongly agree	no answer
information signs	○	○	○	○	○	○
internal information of the department (e.g. meetings, student council, online, personal information transfer)	○	○	○	○	○	○
external information of the department (e.g. university newsletter, webpage)	○	○	○	○	○	○

B2. Please assess whether and to what extent patient care in the dental clinic SINCE the COVID-19 pandemic with respect to the following aspects has changed compared to before the pandemic:

	less	unchanged	more
time required for central patient registration on the first floor (incl. Covid-19 anamnesis)	○	○	○
time required for patient registration in the departments	○	○	○

	less	unchanged	more
waiting time in the waiting rooms of the departments	○	○	○
waiting time for treatment appointments in general	○	○	○
time required for individual treatment sessions	○	○	○

B3. Has your child or yourself had dental treatment postponed due to the COVID-19 pandemic?

- o Yes
- o No

B4. From which side was the above-mentioned appointment postponed?

- o Patient (by yourself)
- o Dental clinic

B5.Why did you postpone the appointment? (Multiple selection possible)

concern about possible infection with COVID-19 in dental clinic	○
family/friends have advised against a visit to the dental clinic due to an increased risk of infection	○
family doctor advised against visiting dental clinic because patient belongs to risk group	○
others	○

B6. What type of appointment was this? (multiple selection possible)

preventive dental appointment/dental check-up	○
prophylaxis/professional tooth cleaning (also as part of supportive periodontal therapy)	○
oral surgical treatment (e.g. tooth extraction, implantation)	○
caries therapy, filling therapy, root canal treatment	○
orthodontic treatment	○
periodontal treatment	○
oral surgery treatment (e.g. tumor treatment)	○
others	○

Was the appointment cancelled without replacement, or if not, by how many weeks?

- o cancelled without replacement
- o …..weeks was the appointment postponed?

C1. To what extent do the following statements apply to you about current patient care during the COVID-19 pandemic?

Please select the appropriate answer for each point:

	strongly disagree	disagree	neutral	agree	strongly agree	No answer
I think it is good that regular patient treatment is maintained with increased hygiene and protective measures and not only emergency treatment is provided.	○	○	○	○	○	○
I am afraid to infect MYSELF.	○	○	○	○	○	○
I am afraid to infect OTHERS.	○	○	○	○	○	○
I feel uncomfortable.	○	○	○	○	○	○
In my opinion, the practitioner feel uncomfortable	○	○	○	○	○	○

C2. How useful do you think are the following measures with regard to protection against infection in the dental clinic/dental treatment?

	strongly disagree	disagree	neutral	agree	strongly agree	no answer
questionnaire to query symptoms and stay in risk areas	○	○	○	○	○	○
wearing mouth-to-nose coverings throughout the whole attendance	○	○	○	○	○	○

	strongly disagree	disagree	neutral	agree	strongly agree	no answer
corona test before each treatment	○	○	○	○	○	○
additional protective equipment (visors, FFP2 masks etc.) on the treatment side	○	○	○	○	○	○
additional hand hygiene						

C3. Which of the following aspects affect you?

	strongly disagree	disagree	neutral	agree	strongly agree	no answer
limited communication due to additional protective equipment (e.g. visor, mask) on the practioners´side	○	○	○	○	○	○
longer waiting time due to additional measures	○	○	○	○	○	○
COVID questionnaire	○	○	○	○	○	○
lack of facial expression due to additional protective equipment (e.g. visor, mask)	○	○	○	○	○	○
waiting time in the outside area of the dental clinic	○	○	○	○	○	○
no toys/books in the waiting area	○	○	○	○	○	○

C4. Were you at least at one time afraid of being infected with COVID-19 at the dental clinic?

- Yes
- No

C5. Please indicate for each month how much fear you had of contracting COVID-19 at the dental clinic.

	Large fear	rather fear	undecided	rather no fear	none fear
mar	○	○	○	○	○
apr	○	○	○	○	○
may	○	○	○	○	○
jun	○	○	○	○	○
jul	○	○	○	○	○
aug	○	○	○	○	○
sep	○	○	○	○	○
oct	○	○	○	○	○
nov	○	○	○	○	○
dec	○	○	○	○	○

C6. Do you think, that all hygiene rules related to infection control were followed in the COVID-19 pandemic in the following areas?

	never	sometimes	often	more often	always
patient registration	○	○	○	○	○
waiting rooms	○	○	○	○	○
treatment /dentists´ room	○	○	○	○	○

C7. Do you perceive the increased hygiene rules and additional protective measures (e.g., wearing mouth/nose covering in the building, hand hygiene, keeping your distance, regular ventilation) as a burden during your visit to the dentist?

- Yes, the measures are a burden for me.
- No, the additional measures are not a problem for me.
- Other

D1. For how long (month/s) do you think the COVID-19 pandemic will continue to change our patient care in the dental clinic?

D2. Would you advocate that the additional protective measures should also be maintained after the COVID-19 pandemic with regard to other airborne infectious diseases (e.g., tuberculosis)?

Yes, always.	○
Yes, depending on the situation (e.g. in the winter months or when there is an increased risk).	○
cannot judge	○
no	○

D3. What additional hygiene and protective measures would you recommend? (Multiple selection possible)

questionnaire to query symptoms and stay in risk areas.	○
one-way street system and entry system	○
wearing of mouth-nose coverings throughout the whole attendance	○
corona test before each treatment	○

additional protective equipment (visors, FFP2 masks, etc.) on the treatment side	○
Structural partitioning of the treatment boxes into individual "treatment rooms"	○
additional hand hygiene	○
others	○

E1. What is your gender or the gender of your child?

Please select only one of the following answers:

- male
- female
- inter / trans

E2. What age group do you or your child belong to?

0- to 4-year-olds	○
5- to 14-year-olds	○
15- to 24-year-olds	○
25- to 34-year-olds	○
35- to 44-year-olds	○
45 to 54-year-olds	○
55- to 64-year-olds	○
65- to 74-year-olds	○
75 to 100-year-olds	○

E3. How many years have you or your child been a patient at the dental clinic?

E4. Do you or your child belong to the risk group with regard to a possible COVID-19 infection?

- o Yes
- o No

E5. Why do you or your child belong to the risk group? (Multiple selection possible)

age +50 years	◯
chronic disease (e.g. diabetes, high blood pressure, heart, lung, liver or kidney disease)	◯
smoker	◯
immunosuppression	◯
overweight	◯
pregnancy	◯
others	◯
