# Supplementary material for: Perspectives from Dentists, Dental Assistants, Students, and Patients on Dental Care Adapted to the COVID-19 Pandemic: A Cross-Sectional Survey
Source: Int J Environ Res Public Health. 2021 Apr 9;18(8):3940. doi: 10.3390/ijerph18083940 (PMC8069116; doi:10.3390/ijerph18083940)
Supplement: Supplementary file 1 [file ijerph-18-03940-s001.zip › Questionnaire students.pdf]

A1 I felt sufficiently informed about the dental care concept adapted to COVID-19 pandemic, by...
Please select the appropriate answer for each point:

	strongly disagree	disagree	neutral	agree	strongly agree	no answer
information signs	○	○	○	○	○	○
internal department information (e.g. meetings, student council, online, personal information transfer meeting)	○	○	○	○	○	○
external department information (e.g. university newsletter, webpage)	○	○	○	○	○	○

A2: Please assess whether and to what extent patient care in the dental clinic SINCE the COVID-19 pandemic with regard to the following aspects has changed compared to before the pandemic:

	less	unchanged	more
time required for admission/exit (incl. changing room)	○	○	○
time required for treatment preparation	○	○	○
waiting time for teachers for tests	○	○	○
time required for individual treatment sessions	○	○	○
number of patient tasks in treatment classes			

B1: To what extent do the following statements apply to you about current patient care during the COVID-19 pandemic?

Please select the appropriate answer for each point:

	strongly disagree	disagree	neutral	agree	strongly agree	no answer
I think it is good that regular patient treatment is maintained with increased hygiene and protective measures and not only emergency treatment is provided.	○	○	○	○	○	○
I am afraid to infect MYSELF.	○	○	○	○	○	○
I am afraid to infect OTHERS.	○	○	○	○	○	○
In my opinion, the patients feel uncomfortable.	○	○	○	○	○	○
I feel uncomfortable.	○	○	○	○	○	○

B2. How useful do you think are the following measures with regard to protection against infection in the dental clinic/dental treatment?

	strongly disagree	disagree	neutral	agree	strongly agree	no answer
questionnaire to query symptoms and stay in risk areas	○	○	○	○	○	○
one-way street and entry system						
wearing mouth-to-nose coverings	○	○	○	○	○	○

throughout the whole attendance						
Corona test before each treatment	○	○	○	○	○	○
additional protective equipment (visors, FFP2 masks etc.) on the treatment side	○	○	○	○	○	○
structural partitioning of the treatment boxes (individual "treatment rooms)	○	○	○	○	○	○

B3. Which of the following aspects affect you?

	strongly disagree	disagree	neutral	agree	strongly agree	no answer
limited communication due to additional protective equipment (e.g. visor, mask)	○	○	○	○	○	○
longer waiting time due to additional measures	○	○	○	○	○	○
lack of facial expression due to additional protective equipment (e.g. visor, mask)	○	○	○	○	○	○
waiting time in the outdoor area of the dental clinic	○	○	○	○	○	○
"traffic jam" at the students´ entrance	○	○	○	○	○	○

B4. Were you at least at one time afraid of being infected with COVID-19 at the dental clinic?

- o Yes
- o No

B5. Please indicate for each month how much fear you had of contracting COVID-19 at the dental clinic.

	Large fear	rather fear	undecided	rather no fear	none fear
mar	○	○	○	○	○
apr	○	○	○	○	○
may	○	○	○	○	○
jun	○	○	○	○	○
jul	○	○	○	○	○
aug	○	○	○	○	○
sep	○	○	○	○	○
oct	○	○	○	○	○
nov	○	○	○	○	○
dec	○	○	○	○	○

B4. Do you think, that all hygiene rules related to infection control were followed in the COVID-19 pandemic in the following areas?

	never	sometimes	often	more often	always
patient registration	○	○	○	○	○
waiting rooms	○	○	○	○	○
treatment /dentists´ room	○	○	○	○	○

B5. Do you perceive the increased hygiene rules and additional protective measures (e.g., wearing mouth/nose covering in the building, hand hygiene, keeping your distance, regular ventilation) as a burden during your visit to the dentist?

- o Yes, the measures are a burden for me.
- o No, the additional measures are not a problem for me.

C1. For how long (month/s) do you think the COVID-19 pandemic will continue to

change our patient care in the dental clinic?

C2. Would you be in favor of maintaining the additional protective measures after the COVID-19 pandemic with regard to other airborne infectious diseases (e.g. tuberculosis)?

Yes, always.	○
Yes, depending on the situation (e.g. in the winter months or when there is an increased risk).	○
I cannot judge.	○
no	○

C3. What additional hygiene and protective measures would you recommend? (Multiple selection possible)

questionnaire to query symptoms and stay in risk areas.	○
one-way street system and admission system	○
wearing of mouth-nose coverings throughout the building	○
corona test before each treatment	○
additional protective equipment (visors, FFP2/N95 masks, etc.) on the treatment side	○
structural partitioning of the treatment boxes into individual "treatment rooms"	○
additional hand hygiene	○
others	○

D1. What is your gender?

Please select only one of the following answers:

- male
- female
- inter / trans

D2. What age group do you or your child belong to?

15- to 24-year-olds	○
25- to 34-year-olds	○
35- to 44-year-olds	○
45 to 54-year-olds	○

55- to 64-year-olds	○
65- to 74-year-olds	○
75 to 100-year-olds	○

D3. In which semester are you currently studying??

7th semester	○
8th semester	○
9th semester	○
10th semester	○
other	○

D4. Do you belong to the risk group with regard to a possible COVID-19 infection?

- Yes
- No

D5. Why do you belong to the risk group? (Multiple selection possible)

age +50 years	○
chronic disease (e.g. diabetes, high blood pressure, heart, lung, liver or kidney disease)	○
smoker	○
immosupression	○
overweight	○
pregnancy	○
others	○

D6. Have you ever been diagnosed with COVID-19?

- o Yes
- o No

D7: Where do you think you became infected with COVID-19?

in private environment (at home)	○
in private environment (vacation)	○
in professional environment (dental clinic)	○
in professional environment (dental praxis)	○
in professional environment (medical appointment)	○
in professional environment (stay in hospital)	○
others	○
